# Acceptability of index partner HIV self-testing among HIV-positive clients in Malawi: A mixed methods analysis

**DOI:** 10.1371/journal.pone.0235008

**Published:** 2020-07-10

**Authors:** O. Agatha Offorjebe, Risa M. Hoffman, Frackson Shaba, Kelvin Balakasi, Dvora Joseph Davey, Mike Nyirenda, Kathryn Dovel

**Affiliations:** 1 Department of Emergency Medicine, Keck School of Medicine, University of Southern California, Los Angeles, CA, United States of America; 2 Division of Infectious Diseases, Department of Medicine, University of California, Los Angeles, CA, United States of America; 3 Partners in Hope, Lilongwe, Malawi; 4 Department of Epidemiology, Fielding School of Public Health, University of California, Los Angeles, CA, United States of America; 5 Division of Epidemiology and Biostatistics, School of Public Health and Family Medicine, University of Cape Town, Cape Town, South Africa; Makerere University School of Public Health, UGANDA

## Abstract

**Objective:**

We sought to evaluate whether HIV-positive adults in Malawi were willing to distribute HIV self-testing (HIVST) kits to their sexual partners of unknown HIV status (index HIVST).

**Design:**

A mixed-methods study was nested within a larger HIVST trial conducted at 15 health facilities in Malawi. Exit surveys were conducted with HIV-positive adults during routine outpatient department visits to assess perceived acceptability of index partner HIVST versus standard partner referral slips that request partner(s) to attend the health facility. Individuals were included in the sub-analysis irrespective of date of HIV diagnosis or ART initiation (or non-initiation). In-depth interviews were conducted with a sub-sample of respondents.

**Results:**

404 HIV-positive adults completed a survey (159 male and 245 female); 21 completed in-depth interviews. Respondents reported feeling more comfortable distributing HIVST versus partner referral slips to their partners (90% vs. 81%) and expressed confidence that their partners would test using HIVST compared to referral slips (77% vs. 66%). Acceptability of HIVST did not vary by sex. Qualitative data revealed that index HIVST was perceived to be private, convenient, and may strengthen relationships by assisting in serostatus disclosure. There were minimal fears of adverse events. Reported barriers to index HIVST included lack of trust within the relationship and harmful gender norms.

**Conclusions:**

HIV-positive clients were willing to distribute HIVST kits to their sexual partners of unknown serostatus. Additional studies are needed to evaluate use of HIVST by index partners, positivity, linkage to care, and adverse events related to index partner HIVST, such as coercion to test among index partners or interpersonal violence among index clients.

## Introduction

Increased testing among partners of individuals living with HIV (index partner testing) is critical to achieving the The Joint United Nations Programme on HIV/AIDS (UNAIDS) 95-95-95 goals in sub-Saharan Africa [1]. Index partner testing is associated with nearly twice the HIV-positivity rates seen with facility-based testing [2] and is a priority testing strategy for reaching epidemic control [3–6]. While overall HIV testing rates have improved, implementation and uptake of index partner testing remains unacceptably poor [7, 8], with male partners significantly less likely to test than female partners [2].

Nearly all index partner testing strategies to date are facility-based, requiring partners to be given a referral slip instructing them to visit the health facility in order to receive testing. Barriers for the HIV-positive client include fear of unwanted disclosure due to distributing an index testing intervention, and fear of adverse events if partners are angry or offended by index partner testing [9–11]. Barriers for the sexual partners of index clients include cost and long travel times to health facilities, long facility wait times and inconvenient testing hours, as well as concerns regarding confidentiality and privacy of testing services [12–15]. Barriers are particularly salient for male partners who have little reason to attend health facilities, have competing demands related to income generation activities, and may be concerned that an HIV-positive status will compromise their status in their community or manhood [16]. Novel, innovative strategies are needed to overcome these barriers.

HIVST provides an outreach approach to address barriers to partner testing, without the logistical and financial burdens often associated with community-based, outreach interventions. HIVST is particularly promising for men who otherwise may not test for HIV[17]. With index partner HIVST, HIV-positive clients enrolled in ART programs can take HIVST kits home to their sexual partners, providing easy access to partners and allowing them to test themselves at their convenience and in the privacy of their own homes [4, 6]. Secondary distribution of HIVST has been shown to be acceptable and feasible among HIV-negative or unknown clients in sub-Saharan Africa [18–21]. Two studies in Kenya found that giving HIVST kits to pregnant/postpartum women and female sex workers led to high levels of secondary distribution to sexual partners [19, 21] and high rates of HIV testing among partners (90%) [19].

However, there is no evidence that index HIVST is acceptable to HIV-positive clients who would be responsible for distributing and explaining the HIVST kit to their partner (secondary distribution). There are real concerns regarding the safety and feasibility of index partner HIVST with HIV-positive individuals. Index partner HIVST may increase fears of unwanted disclosure among HIV-positive clients and may add unrealistic expectations where clients are expected to disclose their HIV status and explain HIVST with their partners, something usually guided by trained, experienced counsellors. This is a critical gap that requires urgent investigation in order to develop appropriate, feasible index partner HIVST strategies. This is particularly critical considering that Ministries of Health throughout sub-Saharan Africa are adopting national policies around index partner HIVST, without experiences or data on how the strategy may work.

We conducted a mixed-methods study to assess the perceived feasibility and acceptability of index partner HIVST by HIV-positive clients versus partner referral slips (standard of care) among HIV-positive clients in Malawi. We included both HIV-positive individuals irrespective of date of HIV diagnosis or ART initiation (or non-initiation). We also examined perceived barriers and facilitators to index partner HIVST.

## Methods

### Ethics statement

Ethical approval was received by the National Health Sciences Research Committee (NHSRC) in Malawi and the Institutional Review Board (IRB) at University of California Los Angeles (UCLA). For the quantitative questionnaire informed, written consent was obtained. For the in-depth interviews, informed, oral consent was obtained. Oral consent was witnessed by research assistants.

### Study design and setting

We conducted cross-sectional surveys and in-depth interviews with HIV-positive individuals at health facilities in central and southern Malawi. Over 9% of the adult population in Malawi are infected with HIV [22]. The Malawian Ministry of Health promotes index partner testing through partner referral slips given to HIV-positive clients for secondary distribution to their index partners. Index partners are then expected to return to the health facility for HIV testing and counselling services. Coverage for index testing is rarely measured, however, a Malawian-based study found that only 22% of index partners referred through this passive partner referral slip system were tested for HIV [7].

### Data collection

Our study was nested within a larger cluster randomized control trial aimed to examine the impact of HIVST on HIV testing uptake among outpatients in high-burden facilities in Malawi (ClinicalTrials.gov identifier NCT03271307 and Pan African Clinical Trials identifier PACTR201711002697316). The trial and its methodology are described in detail elsewhere [23, 24]. Fifteen high-burden facilities participated in the study. Facilities ranged in location (urban vs. rural), size (district hospital vs. rural hospital vs. health center), and by facility type (government vs. mission facilities where HIV care is provided for free, but other provider visits may result in a small fee). See S1 Appendix for a detailed description of facilities. Data were collected between September 2017 –March 2018. Survey tools were piloted with 18-HIV positive individuals attending outpatient departments and refined accordingly.

#### Quantitative data

Exit surveys were conducted with adult outpatient clients (≥15 years of age) immediately following routine outpatient services. Surveys were conducted in the local language (Chichewa) and were completed by trained research assistants in private rooms within the health facility. A total of 5,885 clients were enrolled in the parent study. Our nested study surveyed participants who met the following inclusion criteria: (1) ≥ 15 years of age; (2) ever received an HIV-positive test result; (3) currently in at least one sexual relationship. Individuals were included in this analyses regardless of ART initiation or not. Individuals participating in the index partner testing survey received a demonstration on HIVST using Oraquick HIVST kits[25] and a description of standard Ministry of Health partner referral slips whereby partners are requested to return to the health facility in order to talk to a health care provider (however HIV testing is not mentioned on the slip). Respondents then answered a series of survey questions to assess acceptability and feasibility of index HIVST as compared to standard partner referral slips.

Variables of interest included: (1) client comfort-level with delivery of HIVST or standard partner referral slips to their partner, using a Likert scale from 0 as very uncomfortable to 5 as very comfortable; (2) client report about the likelihood that their partner would test using either HIVST or partner referral slip using the same scale (0–5). For analytic purposes, we collapsed the response categories to dichotomous variables (very uncomfortable/ uncomfortable = 0; very comfortable/comfortable = 1). We also asked participants to choose which intervention (HIVST or partner referral slip) they would prefer distributing, and which intervention they believed their partner would be more likely to use.

We collected data on sociodemographics and relationship status. For analytic purposes, relationship status was measured as a dichotomous variable, married (= 1) and non-married (= 0). We measured literacy instead of educational attainment because literacy is important to reading and understanding both HIVST and partner referral slip strategies for index testing [26, 27]. Further, in Malawi, educational attainment is a poor proxy for literacy levels [28]. Literacy was measured as a dichotomous variable, literate (= 1) or not (= 0), and measured by respondents’ ability to read a brief sentence at a 3^rd^ grade reading level.

Age of respondents, number of children living in the home, and number of current sexual partners were measured as continuous variables. We included a dichotomous variable for clients who were recently diagnosed HIV-positive, defined as tested HIV-positive in the past 3-months (yes/no), because recently diagnosed clients’ may be less comfortable distributing index partner testing materials [9–11].

#### Qualitative data

Anonymous, semi-structured in-depth interviews (IDIs) were conducted with a sub-sample of survey respondents at six health facilities across central and southern Malawi. A random sub-sample of survey respondents who reported ever testing HIV-positive and currently having at least one current sexual partner were recruited for in-depth interviews. All participants completed oral consent before completing the interview. Interviews were conducted by trained interviewers in the local language (Chichewa) who were matched by sex of respondents (male-male, female-female). Interviews assessed barriers and facilitators to index HIVST. All qualitative data were digitally recorded, transcribed, and translated into English for analyses.

### Analyses

#### Quantitative analysis

We used independent t-tests to examine gender differences in the acceptability and feasibility of HIVST and partner referral slips. Since numerous bivariate tests were preformed, we apply a Bonferonni correction to estimate the true probability of a type I [29]. We use multivariable regression analyses to identify predictors of client acceptability for index HIVST. Facility and sex were included in all adjusted models. All other variables that had a p-value<0.1 in the unadjusted models were also included in adjusted models. Results are shown using unadjusted and adjusted odds ratios.

#### Qualitative analysis

Transcripts were coded using deductive codes from the current literature and inductive codes that emerged from the transcripts. OO and KD reviewed the transcripts and applied deductive and inductive codes. Codes were compared for consistency and differences were resolved. Analysis was completed using constant-comparison methods [30] in ATLAS.ti v.1.6 [31]. Dominant themes mentioned across IDIs were extracted and presented below.

## Results

### Quantitative results

404 HIV-positive clients with an active sexual partner completed the index partner testing survey, with 159 (39%) male and 245 (61%) female HIV-positive individuals. Table 1 describes characteristics of respondents. Only 13% of both men and women were newly diagnosed with HIV (within the prior 3 months). Among all participants, 87% of men and 92% of women were currently on ART (irrespective of date of diagnosis). Twenty-six percent of men and 50% of women were illiterate, and 94% of men and 79% of women were married.

**Table 1:** Baseline characteristics of HIV-positive clients with an active sexual partner (n = 404).

Characteristic	Total N = 404	Men n = 159	Women n = 245	p-value*
Mean age, years (IQR)	37.6 (30–44)	42.4 (35–50)	34.4 (27–40)	<0.001
Illiterate, n (%)	164 (41)	42 (26)	122 (50)	<0.001
Married, n (%)	342 (85)	149 (94)	193 (79)	<0.001
Non-married partner, n (%)	62 (15)	10 (6)	52 (21)	<0.001
Mean number of children living in the home (IQR)	3.4 (2–5)	4.1 (2–5)	3 (2–4)	<0.001
Mean number of current sexual partners (IQR)	1.5 (1–2)	2 (1–2)	1.3 (1–1)	<0.001
Diagnosed HIV+ within the past 3-months, n (%)	53 (13)	20 (13)	33 (13)	0.98
Currently on ART, n (%)	365 (90)	139 (87)	226 (92)	0.12
Central Malawi, n (%)	98 (24)	36 (23)	62 (26)	(ref)
Southern Malawi, n (%)	306 (76)	123 (77)	183 (75)	0.79

*p-value calculated using independent t-tests for continuous and chi-square for dichotomous variables

Table 2 describes the feasibility and acceptability of index HIVST by HIV-positive clients versus standard partner referral slips. Clients reported being more comfortable delivering HIVST kits as compared to delivering partner referral slips (90% vs 81%). Clients’ comfort-level to deliver HIVST kits did not differ by sex, while comfort-level for delivering partner referral slips did (male clients: 88% comfortable vs. female clients: 77% comfortable; p-value = 0.007). More clients believed their partners would test using HIVST as compared to return for clinic-based HIV testing following receipt of a partner referral slips (77% vs. 66%). Clients report of their partners’ use of HIVST did not differ by sex, while clients report of partners’ testing with partner referral slips did (male clients: 74% vs female clients: 60%; p = 0.007).

**Table 2:** Acceptability and feasibility of index HIVST by HIV-positive clients vs. partner referral slips by sex (n = 404).

Characteristic	Total (N = 404) n (%)	Men (n = 159) n (%)	Women (n = 245) n (%)	p-value*
Reported comfort distributing the following index testing strategies				
HIV self-test kits	365 (90)	147 (92)	218 (89)	0.27
Partner referral slips	329 (81)	140 (88)	189 (77)	0.009
Prefer distributing self-test kits over partner referral slips	261 (65)	97 (61)	164 (67)	0.31
Believe their primary sex partner would test using the following strategies				
HIV self-test kits	312 (77)	125 (79)	187 (76)	0.59
Partner referral slips	265 (66)	117 (74)	148 (60)	0.009
Believe their primary sex partner would prefer using self-test kits over partner referral slips	256 (63)	104 (65)	152 (62)	0.53

* p-value calculated using independent t-tests for continuous and chi-square for dichotomous variables, Bonferonni correction was applied

Table 3 presents the unadjusted and adjusted odds ratios for HIV-positive clients’ comfort to distribute HIVST or partner referral slips to their sex partners. Married clients (aOR: 2.04, 95% CI = 1.06, 3.93) and those who were literate (aOR: 2.07, 95% CI = 1.09, 3.91) had higher odds of reporting being willing to deliver HIVST to their sexual partners. For partner referral slips, male clients (aOR: 0.1.71, 95% CI = 01.18, 2.93) and married clients (aOR: 0.34, 95% CI = 0.18, 0.64) had higher odds of reporting being willing to deliver partner referral slips to their sexual partners.

**Table 3:** Univariate and multivariable regression analysis of predictors of HIV-positive clients reporting being willing to distribute the testing intervention to their sex partner(s) (n = 404).

	HIVST		Partner Referral Slips	
Characteristic	Unadjusted OR (CI)	Adjusted OR (CI)^+^	Unadjusted OR (CI)	Adjusted OR (CI)^+^
Gender of client				
Male	1.69 (0.87–3.29)	-	**2.10 (1.23–3.58)**	**1.71 (1.18–2.93)**
Female (ref)	-	-	**-**	**-**
Adult (>25years)	1.15 (0.43–3.07)	-	**2.06 (1.02–4.15)**	1.41 (0.67–2.94)
Literate	**2.00 (1.10-.3.67)**	**2.07 (1.09–3.91)**	0.69 (0.42–1.12)	**-**
Diagnosed HIV+ within the past 3-months	0.81 (0.32–2.04)	-	0.98 (0.47–2.05)	-
Enrolled in ART	1.33 (0.53–3.33)	**-**	1.24 (0.59–2.61)	
Married	**2.04 (1.06–3.93**)	**2.04 (1.06–3.93)**	**2.77 (1.69–4.52)**	**2.40 (1.44–4.0)**
Mean number of children living in the home	1.09 (0.92–1.29)	-	0.99 (0.88–1.12)	-
Mean number of current sexual partners	0.97 (0.78–1.20)	-	0.91 (0.78–1.06)	-

Findings with p-value<0.05 are in bold

^+^adjusted for facility and sex and includes variables that had a p-value<0.1 in unadjusted models

### Qualitative results

Twenty-one HIV-positive clients participated in an in-depth interview (13 females and 8 males). Seventeen were married, 1 was separated, and 3 had non-married partners. Differences by gender are highlighted below, although dominant themes were largely similar across gender.

#### Acceptability of index HIVST by HIV-positive clients

Nearly all index clients reported that they were willing to deliver HIVST to their sexual partners. In particular, our qualitative data demonstrates that HIVST is highly acceptable among married couples and couples in stable relationships.

“I have welcomed this tool [HIVST kit] with both hands. I have been trying to tell her [my wife] to get tested so that she should know her HIV status just like I have known mine.” (Male/52/Married)

HIVST was particularly desired by female clients as a strategy to reach their male partners who had previously refused facility-based testing strategies. This is reflected in the quantitative data where female clients were less willing to distribute partner referral slips as compared to HIVST.

“I like it [HIVST] because most of the time I am hesitant, since men think differently from women… When women come here and test HIV positive, they explain to their husband, but men are not open. I am very happy because maybe this is one way that my partner should know his HIV status since he has been refusing to come here [health facility] to get tested.” (Female/47/Married)

Nearly all clients believed their partner would be happy to receive a HIVST kit, with the majority stating that bringing home a kit could strengthen their relationship. A female index client was asked how her relationship might change with delivering a HIVST kit. She responded:

“Yes, it [the relationship] can even get stronger, and not get complicated… because I have been open and explained to him what to do so that his future will be somewhere.” (Female/32/Married)

Clients believed they could demonstrate the kit to their partners, and their partners could use and interpret the HIVST kit. Very few clients believed that additional counselling was needed.

When asked about specific strategies for delivering HIVST, nearly all index clients could describe how they would introduce and explain how to use HIVST to partners, and most believed the way in which they delivered HIVST would influence how their partners received the HIVST kit, and if they would use it. Most clients said HIVST kits should be given in the evening or bedtime, when there are the least distractions.

“In the evening is a good time when we are in bed and we are chatting, so I can be open to explain to him about this way [HIVST].” (Female/32/Married)

“Evening is the best time … During the day there are many things to be preoccupied with… but during evening, we are just the two of us and we are there to make decisions.” (Male/52/Married)

#### Convenience

The majority of index clients believed their partners would test with HIVST because it is convenient. Female clients frequently mentioned that their male partners would not test due to the time required to receive traditional HIV testing.

Respondent: “Currently, he is busy because of farming.”

Interviewer: “So, for him to come here [health facility] instead of farming, he thinks it’s hard?”

Respondent: “Yes, he thinks he will waste time.” (Female/40/Married)

HIVST eliminates time costs associated with traveling to health facilities and waiting for health services.

“I welcome it [HIVST] and feel good because my partner wouldn’t travel the distance to the hospital for an HIV test, but he will just test himself [with HIVST].” (Female/32/Married)

“It will be easy for one to test herself, unlike going to the hospital frequently. So it is better to have HIVST which would make people realize their status in good time [to not delay testing].” (Male/61/Married)

#### Privacy

Some index clients noted that their partners avoided HIV testing because they feared that community members will see them testing and make assumptions about their sexual behaviour.

“Many people fail to test at the facility because they are shy… People are shy [afraid] because when they come here [the facility] and test, they think that people will think that they are prostitutes.” (Male/60/Married)

HIVST alleviates these fears by allowing partners to test in private, assured that community members will not know they sought HIV testing or their test results.

“I have loved this kit because he [my partner] will self-test at home; he is too shy to come to the hospital, which is a public place.” (Female/32/Married)

#### Disclosure

A minority of HIV-positive clients believed HIVST would facilitate status disclosure to their sexual partner because it provides an opportunity to discuss HIV testing.

“It [HIVST] can encourage you to be open to her [partner] and tell her either you have HIV or not.” (Male/19/Non-married Partner)

#### Barriers to index HIVST experienced by HIV-positive clients

The majority of HIV-positive clients did not anticipate major challenges to delivering HIVST. A minority of clients identified lack of trust in the relationship, potential intimate partner violence (IPV), and for male partners, harmful gender norms, such as unequal decision making and male dominance, as potential barriers to index HIVST.

#### Lack of trust

A minority of index clients noted that their sexual partners may question why they brought the HIVST kit home and may become angry. This was especially a concern for couples who already had distrust in the relationship.

“.. but if there isn’t trust [in the relationship] you can’t have the courage to take it [HIVST] to him [partner].” (Female/32/Married)

Others noted that even if the HIVST kit was delivered, partners who did not trust the index client may still refuse testing.

“They would say things like, ‘Did I ask you to get me these? I don’t want it’.”(Female/40/Married)

“If she doesn’t love her life that means she won’t accept the kit, she will say, ‘You don’t trust me’.” (Male/19/Non-Married Partner)

A few index clients said that delivering HIVST could result in the end of the relationship, particularly if results were discordant because partners may assume infidelity by the HIV-positive clients.

“Maybe he [sexual partners] can end the marriage… he can test but be HIV-negative, so he can say, ‘I live with you, yet how can you be positive, and I am negative?’ Then the marriage can end there. Those who can accept that you should still stay [in the relationship] are very few.” (Female/34/Separated)

#### Intimate partner violence

The vast majority of respondents believed that delivering HIVST to partners would not increase risk of IPV.

“There can’t be violence because it will depend on how you explain the thing [HIVST] to him. If you explain everything well there can’t be any violence.” (Female/32/Married)

Only a few clients believed that HIV-positive women may be at risk of IPV upon delivering HIVST. One man described what other male partners may think about a wife bringing home HIVST:

“It is as though the wife has undermined you, but on the contrary, your wife actually wants your life to be well just like she is. But because of short-temperedness, that’s when one [man] can beat his wife.” (Male/52/Married)

An HIV positive-woman also described the potential violence other women could face when delivering the kit:

“If the partner is a man she could even be beaten.” (Female/47/Married)

#### Harmful gender norms

A minority of index clients described that harmful gender norms may impede male partners’ use of HIVST. Some believed that power imbalances within the relationship could deter women from delivering HIVST, while others believed that men are difficult to convince, and may refuse HIVST even if it is delivered to them.

“Problems can be there. I have seen that sometimes the woman goes and gets tested alone, and she goes home and explains [to her husband] that she went to the hospital and got tested. If a woman is to bring back a self-test kit as well, I believe the woman can get 2 or 3 questions about why she took the kit. The man would ask, “What were you thinking by getting this thing!’ So most of the time a woman can be scared to explain, so at the end, the kit will not be given.” (Female/32/Married)

“Based on how I know women, women are not hard to convince. If there is someone who easily accepts what a man says, it’s a woman. But men. If there is someone who will have problems with this tool, it is a man.” (Female/32/Married)

Surprisingly, no one reported concern about discordant test results or negative repercussions for discordant results. However, the vast majority of respondents assumed their partner was HIV-positive removing perceived risk of discordancy.

## Discussion

This is one of the first studies to examine the acceptability of index partner HIVST among HIV-positive clients. Both quantitative and qualitative data found that index partner HIVST was reported to be highly acceptable among HIV-positive clients. HIV-positive clients reported being more comfortable distributing HIVST kits as compared to partner referral slips and believed their partners would also be more likely to test with HIVST than come to the health facility for HIV testing. Findings are consistent with other HIVST studies examining secondary distribution by HIV-negative or unknown clients [18, 19, 21]. Notably, our qualitative results show that index HIVST distributed by HIV-positive clients may increase testing among male partners by addressing traditional barriers to men’s testing, including inconvenience of test location, time and transport costs associated with facility-based services, and fear of unwanted disclosure and stigma [13, 15].

HIV-positive clients believed their partners would be willing to use HIVST because HIVST is convenient and private. Similar findings have been found for multiple HIVST distribution strategies [32–34]. Secondary distribution studies in Kenya show dramatic increases in testing coverage among sexual partners of pregnant/postpartum women and female sex workers who were given HIVST kits [19, 21], with little to know adverse events reported. A novel finding from our study is that HIV-positive clients believed HIVST may strengthen relationships by assisting with disclosure and creating an environment for discussing HIV and HIV services as a couple. Other literature finds that disclosure may strengthen relationships [35–37]. HIVST as a tool for disclosure is a novel finding that warrants further exploration.

Although index partner HIVST was reported to be highly acceptable among HIV-positive clients, several barriers may limit this strategy. Similar to partner referral slips, unmarried clients were less likely to be willing to distribute HIVST. Importantly, illiterate clients were also less likely to be willing to distribute HIVST, reflecting a potential gap in the types of populations that can be reached with secondary HIVST strategies. Similar to other research, a minority of HIV-positive clients noted that lack of trust in the relationship [11, 37, 38] and harmful gender norms [37, 38] may discourage HIVST distribution and use. While index partner HIVST may improve access and use of HIV testing, particularly among male partners, it may still face barriers related to couple dynamics, as seen in other couples-based HIV services.

Notably, IPV [36–38] was only mentioned by a few respondents, and many felt that adverse events may be prevented based on how HIVST is introduced to sexual partners, such as delivering the intervention in a private setting with limited distractions, similar to other studies with HIV-unknown/negative clients [39]. Risk of IPV may be particularly high for index clients whose partner tests HIV-negative, as disclosure and sero-discordancy are shown to increase violence experienced among HIV-positive women [40]. However, nearly all respondents in in-depth interviews believed that their partner was HIV-positive, and therefore had little concern regarding potential discordancy. This may reflect general practices throughout the region whereby individuals assume they have the same serostatus as their primary partner. Further research is needed to understand actual risk of IPV among HIV-positive clients, and if index HIVST is feasible for unmarried couples, those with mistrust in the relationship, and male partners who hold harmful notions of gender norms.

The study had several limitations. First, this was a hypothetical scenario. HIV-positive clients were not actually given HIVST kits, and thus findings are based on perceived acceptability. However, we provided a HIVST demonstration to all participants in order to increase familiarity with HIVST. Second, the study was conducted in Malawi and may not be generalizable outside Malawi settings, or among HIV positive individuals who are not actively engaged in ART care. Further, our sample was older and findings may differ for young, unmarried individuals. Additional research is needed on acceptability of partner index HIVST among young, unstable partnerships and contexts with higher levels of IPV and fluid relationships. Finally, our study was a sub-study of a parent study powered to evaluate the effect of facility HIV self-testing on same-day HIV testing among outpatient clients in Malawi. As a result, this sub-study may be under-powered to evaluate the potential acceptability of HIVST in this population as we used the sample required for the larger study.

## Conclusion

Index partner HIVST is a promising strategy for improving index partner testing in sub-Saharan Africa. HIV-positive clients believed index partner HIVST was an acceptable and feasible, with minimal risk of adverse events. Importantly, index partner HIVST may address traditional gender-based barriers to testing for men by making testing accessible and private. Future studies are needed to examine actual distribution, use, adverse events related to index partner HIVST, and linkage to care.

## Supporting information

S1 Appendix (DOCX)
