# Supplementary material for: Acceptability of index partner HIV self-testing among HIV-positive clients in Malawi: A mixed methods analysis
Source: PLoS One. 2020 Jul 10;15(7):e0235008. doi: 10.1371/journal.pone.0235008 (PMC7351183; doi:10.1371/journal.pone.0235008)
Supplement: S1 Appendix — (DOCX) [file pone.0235008.s001.docx]

**Appendix A.**

**Table 4. Facility level characteristics (n=15).**

|  | **Southern Region** | **Central Region** | **Total** |
| --- | --- | --- | --- |
|  | n (%) | n (%) | n (%) |
| **Total facilities** | 8 | 7 | 15 |
| **HIV services** |  |  |  |
| HIV services integrated within outpatient |  |  |  |
| Department | 0 (0) | 0 (0) | 0 (0) |
| Same day ART initiation | 8 (100) | 7 (100) | 15 (100) |
| **Facility type** |  |  |  |
| Hospital | 2 (25%) | 1 (14%) | 3 (20%) |
| Mission hospital | 1 (13%) | 2 (29%) | 3 (20%) |
| Health facility | 5 (62%) | 4 (57%) | 9 (60%) |
